# Calcium Microdomain Formation at the Perisynaptic Cradle Due to NCX Reversal: A Computational Study

**DOI:** 10.3389/fncel.2019.00185

**Published:** 2019-05-07

**Authors:** John Joseph Wade, Kevin Breslin, KongFatt Wong-Lin, Jim Harkin, Bronac Flanagan, Harm Van Zalinge, Steve Hall, Mark Dallas, Angela Bithell, Alexei Verkhratsky, Liam McDaid

**Affiliations:** ^1^Computational Neuroscience and Neural Engineering (CNET) Research Team, Intelligent Systems Research Centre, Ulster University, Derry, United Kingdom; ^2^Neural Systems and Neurotechnology Research Team, Intelligent Systems Research Centre, Ulster University, Derry, United Kingdom; ^3^Department of Electrical Engineering and Electronics, University of Liverpool, Liverpool, United Kingdom; ^4^Reading School of Pharmacy, University of Reading, Reading, United Kingdom; ^5^Faculty of Biology, Medicine and Health, University of Manchester, Manchester, United Kingdom; ^6^Achucarro Center for Neuroscience, IKERBASQUE, Basque Foundation for Science, Bilbao, Spain

**Keywords:** perisynaptic cradle, calcium microdomains, astrocytic process, Na^+^/Ca^2+^ exchange, compartment model, glutamate transport, sodium dynamics

## Abstract

It has recently been proposed using a multi-compartmental mathematical model that negatively fixed charged membrane-associated sites constrain the flow of cations in perisynaptic astroglial processes. This restricted movement of ions between the perisynaptic cradle (PsC), principal astroglial processes and the astrocyte soma gives rise to potassium (K^+^) and sodium (Na^+^) microdomains at the PsC. The present paper extends the above model to demonstrate that the formation of an Na^+^ microdomain can reverse the Na^+^/Ca^2+^ exchanger (NCX) thus providing an additional source of calcium (Ca^2+^) at the PsC. Results presented clearly show that reversal of the Na^+^/Ca^2+^ exchanger is instigated by a glutamate transporter coupled increase in concentration of cytoplasmic [Na^+^]_i_ at the PsC, which and instigates Ca^2+^ influx through the NCX. As the flow of Ca^2+^ along the astrocyte process and away from the PsC is also constrained by Ca^2+^ binding proteins, then a Ca^2+^ microdomain forms at the PsC. The paper also serves to demonstrate that the EAAT, NKA, and NCX represent the minimal requirement necessary and sufficient for the development of a Ca^2+^ microdomain and that these mechanisms directly link neuronal activity and glutamate release to the formation of localized Na^+^ and Ca^2+^ microdomains signals at the PsC. This local source of Ca^2+^ can provide a previously underexplored form of astroglial Ca^2+^ signaling.

## Introduction

The concept of astroglial ionic excitability was established in 1990s following the discovery of calcium ion (Ca^2+^) signaling and propagating Ca^2+^ waves in astrocytes *in vitro* and *in situ* (Cornell-Bell et al., 1990; Cornell-Bell and Finkbeiner, 1991; Dani et al., 1992; Verkhratsky et al., 1998). Initially, astroglial excitability has been thought to be mediated by Ca^2+^ ions; subsequent years have demonstrated signaling roles for sodium (Na^+^) (Kirischuk et al., 2012; Parpura and Verkhratsky, 2012; Rose and Chatton, 2016; Rose and Verkhratsky, 2016) and chloride (Cl^−^) (Wilson and Mongin, 2018) while a signaling role for potassium (K^+^) has also begun to be considered (Breslin et al., 2018), see also (Verkhratsky and Nedergaard, 2018). Ionic signaling in astrocytes serves several physiological roles, in particular, coupling neuronal activity with astroglial homeostatic response within the confines of the astroglial cradle that enwraps at least 50% of all synapses in the central nervous system (Verkhratsky and Nedergaard, 2014, 2018; Rose and Verkhratsky, 2016). Astrocytes express elaborate molecular machinery controlling sodium (Na^+^) homeostasis and allowing transient intracellular Na^+^ increases in response to physiological activity in neuronal networks (Kirischuk et al., 2012; Rose and Verkhratsky, 2016). The Na^+^ signals localized to perisynaptic astroglial processes regulate the activity of numerous plasmalemmal transporters responsible for a glutamine-glutamate (GABA) astroglial-neuronal shuttle, for K^+^ buffering, for regulation of pH and for cellular metabolism as well as for secretion of reactive oxygen species scavengers and various neuroactive molecules (Rose and Verkhratsky, 2016; Verkhratsky and Nedergaard, 2016, 2018). Cytoplasmic Na^+^ dynamics are therefore directly linked to the functional activity of astrocytes and represent a mechanism for fast and local signaling at the single synapse/perisynaptic process level. Although cytosolic Na^+^ has emerged as a prominent ion at the interface between signaling and metabolic pathways (Chatton et al., 2016), the spatiotemporal organization of cytosolic Na^+^ dynamics is far from being fully characterized. Additionally, pathways that serve to trigger Na^+^ and K^+^ microdomains need to be resolved.

Computational modeling of astrocyte-neurone interaction is essential for the understanding of the transport processes between cells. However, models of ionic signaling in astrocytes still have some way to go if they are to fully capture the complex morphology of astrocytic processes seen *in vivo*. Some of these models focus on changes of ionic fluxes crossing the astrocyte membrane controlled by pumps and exchangers, for example Na^+^/K^+^ pump (NKA) and Na^+^/Ca^2+^ exchanger, (NCX) and K^+^, Na^+^ and Cl^−^ channels as well as axial fluxes of ions in the intracellular space (Halnes et al., 2013). This electro-diffusive model allows channels to be distributed in different ways while gating parameters can be altered to match experimental data. Intercellular Ca^2+^ waves and oscillations in astrocytes (Pasti et al., 1997; Wade et al., 2011, 2012; Naeem et al., 2015) have also been modeled to identify potential mechanisms of neurone to astrocyte intracellular intercellular signaling.

### Astrocytic Na^+^ Signaling

The major contributors to astrocyte Na^+^ homeostasis and signaling are the NKA, NCX and the sodium-dependent glutamate transporters (EAAT1 and EAAT2). The NKA regulates resting Na^+^ concentration in the cytosol ([Na^+^]_i_) and expels excess Na^+^ that enters astrocytes during periods of neuronal activity, whereas glutamate transporters are responsible for the bulk of Na^+^ influx into astrocytes accompanying glutamatergic synaptic transmission (Kirischuk et al., 2007; Rose and Karus, 2013; Rose and Verkhratsky, 2016). The NKA, NCX, and glutamate transporters co-localize in astrocytic perisynaptic processes (Minelli et al., 2007; Melone et al., 2018), suggesting their functional coupling.

All three subunits of NCX (NCX1/SLC8A1, NCX2/SLC8A2, and NCX3/SLC8A3) are expressed in astroglia, with NCX1/SLC8A1 being the predominant isoform (Pappalardo et al., 2014; Verkhratsky and Nedergaard, 2018). The stoichiometry of astroglial NCX is 3Na^+^:1Ca^2+^, and hence the equilibrium potential can be calculated from Nernst equation: E_NCX_ = (nE_Na_−2E_Ca_)/(*n*−2) where n is a stoichiometry of Na^+^, and E_Na_ and E_Ca_ are equilibrium potentials of Na^+^ and Ca^2+^, respectively. Assuming [Ca^2+^]_i_ of 50–80 nM and [Na^+^]_i_ of 15 mM, the E_NCX_ could be as negative as ~−85 to −90 mV, in a similar range to measured resting membrane potential values of astrocytes (Verkhratsky and Nedergaard, 2018). As a result the NCX is prone to fluctuate between forward and reverse transport depending on actual changes in [Ca^2+^]_i_ and [Na^+^]_i_ and the astroglial membrane potential (Vm). Conceptually, depolarization or an increase in [Na^+^]i will favor NCX operation in the reverse mode, whereas an increase in [Ca^2+^]i promotes the forward mode of the exchanger. In this way NCX can regulate both Ca^2+^ and Na^+^ signals, being relevant in shaping ionic signals in astroglial PsCs.

Recently we presented a new hypothesis (Breslin et al., 2018), which addressed ionic dynamics in thin (<100 nm) perisynaptic processes whereby negatively charged lipids form deep potential wells near the dipole heads restricting the flow of cations along the process. The negative ion foci serve to form “traps” that attract free cytosolic cations forcing them to hop from trap to trap, thus restricting ion propagation along the process and isolating them from the soma. This “ionic retention by traps” can potentially explain the generation of the transient Na^+^ and K^+^ microdomains at PsCs. The present paper aims to further develop this model with the inclusion of Ca^2+^ dynamics at the PsC. We stress here that while this model is not the focus of this paper, it is necessary to include it as it replicates closely the experimentally observed Na^+^ microdomain in astroglial processes (Langer and Rose, 2009). Furthermore, the model allows us to test the hypothesis that reversal of the NCX, due to the Na^+^ microdomain, leads to Ca^2+^ influx and this, coupled with Ca^2+^ retention along the thin astrocyte process, provides for a plausible mechanism for the generation of a Ca^2+^ microdomain at the PsC. Moreover, as this source of Ca^2+^ is remote from any endoplasmic reticulum (ER) mediated Ca^2+^ release, we propose that this local source of Ca^2+^ may provide a previously under-explored form of astrocyte Ca^2+^ signaling.

## Model

**Figure 1:**
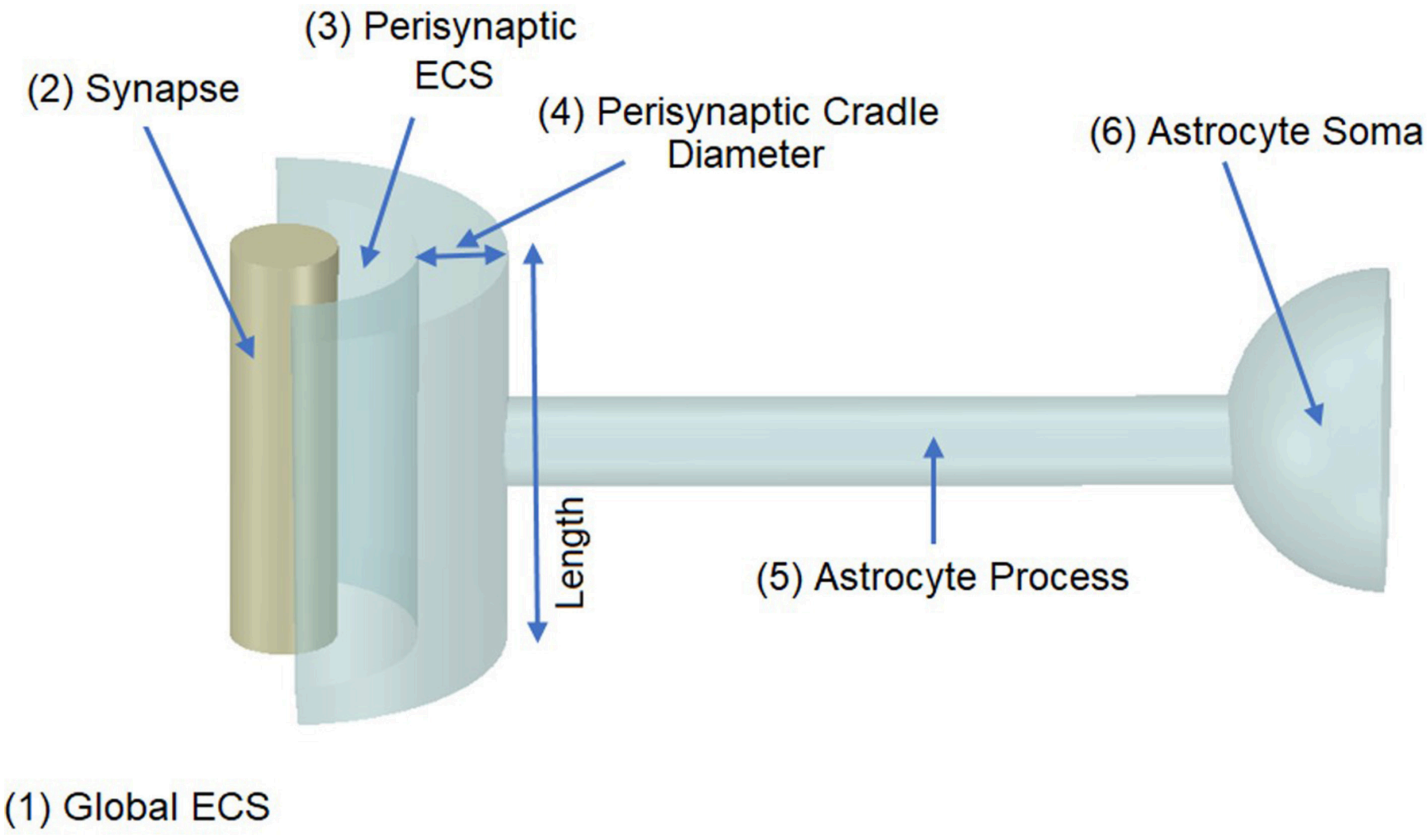
Model Morphology. The model consists of a single synapse enwrapped by a single astrocyte. In total there are six compartments, (1) Global Extracellular Space (GECS), (2) Synapse, (3) Perisynaptic Extracellular Space (PsECS), (4) Perisynaptic Cradle, (5) Astrocyte Process, and (6) Astrocyte Soma. Each compartment is modeled as a cylindrical structure except the GECS and soma, which are deemed dimensionless because ionic concentrations remain constant within these compartments (Breslin et al., 2018).

It was proposed in our earlier paper (Breslin et al., 2018) that Na^+^ and K^+^ retention occurs in thin astrocyte processes. In this paper we consider Ca^2+^ retention along the thin processes. This Ca^2+^ retention is captured using an extended form of the multi-compartmental mathematical model described in Breslin et al. (2018). The model consists of a single synapse surrounded by an astrocytic PsC. Due to the complexity of neuronal/astrocytic morphology (Xu-Friedman et al., 2001; Witcher et al., 2007; Lushnikova et al., 2009; Patrushev et al., 2013), this simplified cylindrical compartmental model (Breslin et al., 2018) simplifies the highly complex structures and the associated computational overheads, whilst retaining sufficient functionality to produce meaningful observations. All necessary dimensional details of the model can be found in Table 1. Figure 1 provides details of the multi-compartmental model described by Breslin et al. (2018).

**Table 1:** Astrocyte morphology.

**Parameter**	**Value**	**Units**	**Description**
**LENGTHS**			
d_IPS_	300 × 10^−9^	m	Perisynaptic internal diameter
d_EPS_	500 × 10^−9^	m	Perisynaptic external diameter
r_IPS_	150 × 10^−9^	m	Perisynaptic internal radius
r_EPS_	250 × 10^−9^	m	Perisynaptic external radius
l_PS_	300 × 10^−9^	m	Perisynaptic length
d_P_	100 × 10^−9^	m	Process diameter
r_P_	50 × 10^−9^	m	Process radius
l_P_	25 × 10^−6^	m	Process length
d_Syn_	270 × 10^−9^	m	Synapse diameter
r_Syn_	135 × 10^−9^	m	Synapse radius
l_syn_	300 × 10^−9^	m	Synapse length
**AREAS**			
CSA_PS_	3.5343 × 10^−14^	m^2^	Perisynaptic cross sectional area
SA_PS_	1.4137 × 10^−13^	m^2^	Perisynaptic surface area
CSA_P_	7.854 × 10^−15^	m^2^	Process cross sectional area
SA_P_	7.854 × 10^−12^	m^2^	Process surface area
CSA_Syn_	2.8628 × 10^−14^	m^2^	Synapse cross sectional area
SA_Syn_	1.2723 × 10^−13^	m^2^	Synapse surface area
SA_PsECS−GECS_	1.5715 × 10^−14^	m^2^	Surface area between PsECS and GECS
**VOLUMES**			
Vol_PS_	1.8850 × 10^−17^	L	Perisynaptic volume
Vol_P_	1.9635 × 10^−16^	L	Process volume
Vol_Syn_	8.5883 × 10^−16^	L	Synapse volume
Vol_PsECS_	2.0145 × 10^−18^	L	Perisynaptic ECS volume

**Figure 2:**
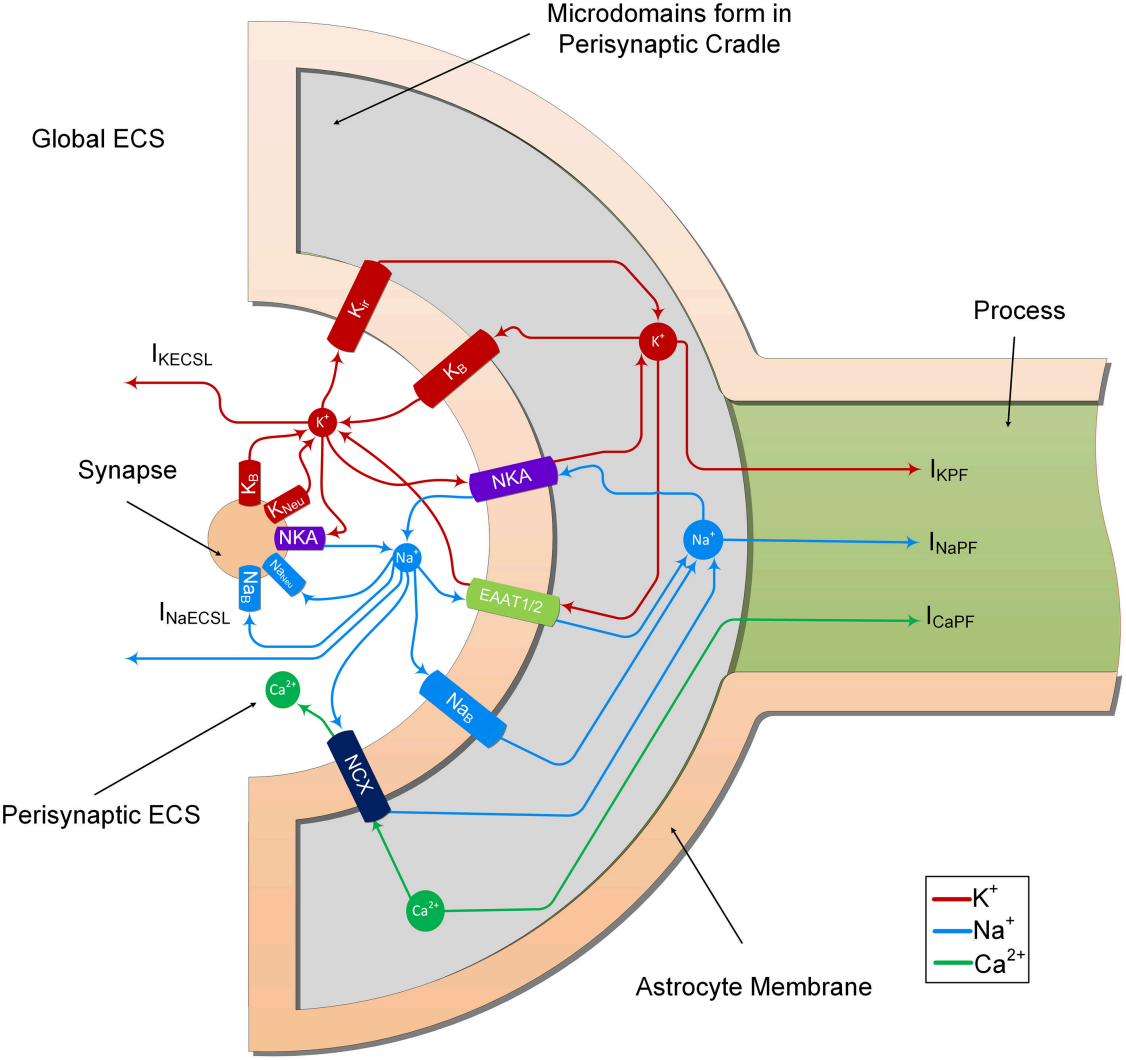
Ion transport machinery of the perisynaptic cradle and synapse. The model consists of 11 ionic transports. The synapse contains 5 ionic transports for Na^+^ and K^+^; NKA is the sodium/potassium pump which extrudes 3 Na^+^ ions for every 2 K^+^ ions it imports, Na_neu_ and K_neu_ which are the voltage gated sodium and potassium channels of the Hodgkin and Huxley model and Na_B_ and K_B_ which are lump models of all the other Na^+^ and K^+^ channels. The astrocyte contains 6 channels on the inside surface area (facing the synapse) of the perisynaptic cradle; Kir is an inward rectifying K^+^ channel, NKA is a sodium potassium pump similar to that found on the synapse, EAAT1/2 represents the EAAT glutamate transport which is sensitive to glutamate in the synaptic cleft. This transport cotransports 3 Na^+^ ions with every glutamate ion and counter transports 1 K^+^ ion. Since this work does not consider changes in synaptic and perisynaptic glutamate changes the glutamate ion is not shown in the figure. Moreover, the EAAT dependency on H^+^ is also ignored. The NCX represents the sodium/calcium exchanger which exchanges 3 Na^+^ ions for every 1 Ca^2+^ ion across the membrane. At resting conditions there is a very small exchange in the forward direction as noted in the diagram. This can be reversed under physiological increase of perisynaptic Na^+^ concentration. Finally, the Na_B_ and K_B_ are lumped models of all other Na^+^ and K^+^ ionic transports, respectively. The currents I_KECSL_ and I_NaECSL_ represent leak currents of K^+^ and Na^+^, respectively from the Perisynaptic Extracellular Space (ECS) to the global ECS. It is worth nothing that the currents I_KPF_, I_NaPF_, and I_CaPF_ represent the currents of the 3 ions under consideration from the perisynaptic cradle along the process. As described in Breslin et al. (2018), these currents model the hypothesized well-hopping mechanism which severely restricts current flow of these ions along thin processes. This results in the trapping of these ions in the perisynaptic cradle as they are imported across the membrane. Since our model assumes a well-mixed compartment, we consider these trapped ions as a microdomain formation in the perisynaptic cradle.

In the previous study (Breslin et al., 2018), K^+^ and Na^+^ were modeled to determine their influence on ion retention in the thin astrocyte process. In the current study, we adapt this model to include Ca^2+^ dynamics within the astrocyte PsC and extracellular space. From Figure 2, it can be seen that the synapse and PsC contain a number of ionic channels, exchangers and pumps to provide homeostasis and dynamic exchange of ions between the two cells and extracellular space.

The neurone exchanges K^+^ and Na^+^ with the PsECS via a voltage-gated potassium channel (K_Neu_), voltage-gated sodium channel (Na_Neu_), a sodium potassium pump (NKA_Neu_), a potassium background channel (K_B_ on the synapse), and a sodium background channel (Na_B_ on the synapse). The astrocyte exchanges Na^+^, K^+^, and Ca^2+^ with the PsECS via a background sodium transport (Na_B_ on the astrocyte), potassium background transport (K_B_ on the astrocyte), potassium inwardly rectifying channel (K_ir_), sodium-potassium-ATPase (NKA), a glutamate-sodium-potassium-proton antiporter (EAAT1/2), and the NCX. In both the astrocyte and neurone models, we define Na^+^ and K^+^ background channels; although modeled as a single transport channel for each ion, these background channels represent a lumped model for Na^+^ and K^+^ transport, taking into account a multitude of influx and efflux pathways. I_KPF_, I_NaPF_, and I_CaPF_ model the flow of K^+^, Na^+^, and Ca^2+^ ions along the astrocytic process to the soma. I_KECSL_ and I_NaECSL_ models the K^+^ and Na^+^ ionic flow generated by K^+^ and Na^+^ leaking from the PsECS to the GECS. The mathematical descriptions of ionic exchanges between a neurone and astrocyte at the PsC are now presented. For a more detailed description refer to Breslin et al. (2018).

### Astrocyte Model

#### Membrane Potential and Ion Concentrations

In this model the astrocyte resting membrane potential is taken as ~−80 mV, which is widely reported as the resting membrane potential for astrocytes (Verkhratsky and Nedergaard, 2018). We also assume that isopotentiality is preserved in the PsC as is the case for the astroglial syncytium (Kofuji and Newman, 2004), therefore the astrocyte membrane potential remains fixed at ~−80 mV.

The perisynaptic model described by Breslin et al. (2018) comprises five compartments, namely PsC, PsECS, GECS, and the astrocyte process and soma. The astrocyte process is modeled as a long thin cylindrical channel that restricts the flow of cations along the process within the channel due to ion retention. In this work, each of these compartments contains three ionic concentrations, K^+^, Na^+^, and Ca^2+^. All channels, exchangers and transporters permeable to these ions reside on the PsC. The kinetic equations for the changes of ionic concentration of each of these ions is given below. Note: *z*_*x*_*FVol*_*y*_ is used to convert the total ionic current of ion *x* into a concentration for the volume *y*, where z_x_ is the valency of ion x, F is Faradays constant, and Vol_y_ is the volume of compartment y. All initial conditions and parameters for this model are described in Tables 2, 3, respectively. The change in PsC K^+^ concentration ([K^+^]_PsC_) in the PsC is given by:

$$\begin{array}{l} \frac{{d\left[K^{+}\right]}_{PsC}}{dt}=\;-\left(\frac{I_{Kir}+I_{KNKA}+I_{KEAAT}{+I}_{KPF}}{z_{K}FVol_{PsC}}\right) \end{array}$$

where *I*_*Kir*_ is the K_ir_ channel current, *I*_*KNKA*_ is the K^+^ current through the astrocyte NKA, *I*_*KEAAT*_ is the K^+^ current created by the glutamate transporter and *I*_*KPF*_ is the K^+^ current flowing along the astrocyte process. K^+^ changes in the PsECS ([*K*^+^]_PsECS_) is given by:

$$\begin{array}{l} \frac{{d\left[K^{+}\right]}_{PsECS}}{dt}=\;-\left(\frac{I_{KECSL}+I_{KNeu}-I_{Km}}{z_{K}FVol_{PsECS}}\right) \end{array}$$

where *I*_*KECSL*_ is current due to K^+^ leakage from the PsECS to the GECS, *I*_*KNeu*_ is the K^+^ current from the neurone, and *I*_*Km*_ is the total K^+^ current flowing through the astrocyte membrane. *K*^+^ is held constant at baseline in the GECS and astrocyte soma compartments.

**Table 2:** Astrocyte model variables.

**Variable**	**Initial Value**	**Units**	**Description**
V_A_	−0.0807	V	Astrocyte membrane potential
[${\text{K}}^{+}{]}_{\text{PsC}}$	0.1	M	K^+^ concentration in the perisynaptic cradle
[${\text{Na}}^{+}{]}_{\text{PsC}}$	0.015	M	Na^+^ concentration in the perisynaptic cradle
[${\text{K}}^{+}{]}_{\text{PsECS}}$	0.004	M	Perisynaptic extracellular K^+^ concentration
[${\text{Na}}^{+}{]}_{\text{PsECs}}$	0.135	M	Perisynaptic extracellular Na^+^ concentration
[Glu]_ECS_	25 × 10^−9^	M	Perisynaptic extracellular glutamate concentration

**Table 3:** Astrocyte model parameters.

**Parameter**	**Value**	**Units**	**Description**
V_m_	−0.0807	V	Astrocyte resting membrane potential
ϕ_w_	0.267	eV	Well activation energy
k_B_	1.38 × 10^−23^	J/K	Boltzmann constant
R	8.31	J/mol/K	Gas constant
T	310	K	Temperature
F	96,485	C/mol	Faraday constant
Q	1.6022 × 10^−19^	C	Coulomb
C_m_	0.01	F/m^2^	Membrane capacitance
g_Kir_	144	S/m^2^	K_ir_ channel conductance
g_K_	17.9364	S/m^2^	K^+^ background transport conductance
g_Na_	0.9761	S/m^2^	Na^+^ background transport conductance
K_K_	0.018	S/m	K^+^ Poole-Frenkel channel constant
K_Na_	0.018	S/m	Na^+^ Poole-Frenkel channel constant
PNKA_max_	0.1 × 10^−5^	mol/m^2^	Maximum NKA-ATPase Pump Rate
K_Nai_	10 × 10^−3^	M	Na^+^ threshold for NKA-ATPase
K_KE_	1.5 × 10^−3^	M	K^+^ threshold for NKA-ATPase
z_K_	1		K^+^ valency
z_Na_	1		Na^+^ valency
z_Ca_	2		Calcium valency
[H^+^]_PsC_	60 × 10^−9^	M	H^+^ Concentration in the perisynaptic cradle
[Glu]_PsC_	1.5 × 10^−3^	M	Glutamate concentration in the perisynaptic cradle
[K^+^]_AS_	0.1	M	K^+^ concentration in the astrocyte soma
[Na^+^]_AS_	0.015	M	Na^+^ concentration in the astrocyte soma
[Ca^2+^]_AS_	100 × 10^−9^	M	Ca^2+^ concentration in the astrocyte soma
[H^+^]_PsECS_	40 × 10^−9^	M	Perisynaptic extracellular H^+^ concentration
[Ca^2+^]_PsECS_	1.5 × 10^−3^	M	Perisynaptic extracellular Ca^2+^ concentration
[K^+^]_GECS_	0.004	M	Perisynaptic global ECS K^+^ concentration
[Na^+^]_GECS_	0.135	M	Perisynaptic global ECS Na^+^ concentration
[Ca^2+^]_GECS_	1.5 × 10^−3^	M	Perisynaptic global ECS Ca^2+^ concentration
[Ca^2+^]_PsC_	100 × 10^−9^	M	Ca^2+^ concentration in the perisynaptic cradle
[Ca^2+^]_PsECS_	1.5 × 10^−3^	M	Perisynaptic extracellular Ca^2+^ concentration
ε_0_	8.85 × 10^−12^	F/m	Vacuum permittivity
ε_r_	0.82	F/m	Relative permittivity of brain tissue
g_ECS_	3.3	S/m^2^	Perisynaptic ECS leak conductance
*α_*EAAT*_*	0.0032	A/m^2^	Glutamate transport fitting parameter
*β_*EAAT*_*	28.8	mV^−1^	Glutamate transport fitting parameter
*r_*g*_*	5 × 10^−7^	M^−1^	Slope of glutamate uptake
*s_*g*_*	9 × 10^−6^	M	Threshold for glutamate uptake
${\bar{I}}_{NCX}$	1	A/m^2^	
Γ	0.5		NCX partition parameter
*J_0_*	0.06	M/s	Maximum EAAT1/2 Flux rate

Changes in the PsC Na^+^ concentration ([*Na*^+^]_PsC_) is given by:

$$\begin{array}{l} \frac{{d\left[Na^{+}\right]}_{PsC}}{dt}=\;-\left(\frac{I_{NaB}+I_{NaNKA}+I_{NaEAAT}+{I_{NaNCX}+I}_{NaPF}}{z_{Na}FVol_{PsC}}\right) \end{array}$$

where *I*_*NaB*_ is a current due to Na^+^ influx across the membrane via Na^+^ permeable ion channels, this is referred to as background *Na*^+^ channel (Breslin et al., 2018), *I*_*NaNKA*_ is the Na^+^ dependent current component of the astrocyte NKA, *I*_*NaEAAT*_ is the Na^+^ current component of the glutamate transporter, *I*_*NaNCX*_ is the Na^+^ current component of the NCX and *I*_*NaPF*_ is the Na^+^ current flowing in the astrocyte process. [Na^+^] changes in the PsECS ([*Na*^+^]_PsECS_) is given by:

$$\begin{array}{l} \frac{{d\left[Na^{+}\right]}_{PsECS}}{dt}=\;-\left(\frac{I_{NaECSL}+I_{NaNeu}-I_{Nam}}{z_{K}FVol_{PsECS}}\right) \end{array}$$

where *I*_*NaECSL*_ is current due to Na^+^ leakage from the PsECS to the GECS, *I*_*NaNeu*_ is the Na^+^ current from the neurone and *I*_*Nam*_ is the total Na^+^ current flowing through the astrocyte membrane. Na^+^ is held constant at baseline in the GECS and astrocyte soma compartments.

Changes in the PsC Ca^2+^ concentration ([*Ca*^+^]_PsC_) is given by:

$$\begin{array}{l} \frac{{d\left[Ca^{2+}\right]}_{PsC}}{dt}=\;-\left(\frac{I_{CaNCx}+I_{CaPF}}{z_{Ca}FVol_{PsC}}\right) \end{array}$$

where *I*_*CaNCX*_ is the Ca^2+^ dependent current component of the NCX, and *I*_*CaPF*_ is the Ca^2+^ current flowing in the astrocyte process. [Ca^2+^] changes in all other compartments are not considered and remain constant at baseline.

#### Glutamate Transporter (EAAT1/2)

Glutamate released into the extracellular PsECS in the course of neurotransmission is assumed here to be entirely removed by astrocytic EAAT1/2. A transport cycle involves the co-transport of 3 Na^+^ and 1 H^+^ with 1 glutamate and counter-transport of 1 K^+^ (Grewer et al., 2014; Murphy-Royal et al., 2015). EAAT1/2 proteins are trafficked to the plasma membrane to facilitate the rapid removal (~3 ms) of glutamate from the cleft. Glutamate bound to these proteins is then transported to the astrocytic cytosol over a longer period: in this work a complete transport cycle is assumed to be 30 ms (Otis and Kavanaugh, 2000; Zhou and Danbolt, 2013). Existing EAAT mathematical formulations do not capture adequately this rapid binding and slow release function and therefore in this work we adopt a different approach. To model the stoichiometry and cycle rate we assume that initially a release of glutamate instantaneously binds to membrane-bound proteins and thereafter the flux of Na^+^ through the EAAT transporter pore follows an exponentially decaying rate given by:

$$\begin{array}{l} \frac{dJ_{NaEAAT}}{dt}=-\;\frac{J_{NaEAAT}\left(t\right)}{\tau }+\;J_{0}\delta \left(t-tsp\right) \end{array}$$

where *J*_*NaEAAT*_ is the flux rate of *Na*^+^ through the EAAT1/2, *Jo* is the max flux rate through the transporter, δ is the Dirac Delta function, *t* is time, and *tsp* is the previous neuronal spike time. In our model we view the membrane as a capacitor charged with bound glutamate and *J*_*NaEAAT*_ as a discharging flux.

The Na^+^ current through the transporter can be calculated by:

$$\begin{array}{l} I_{NaEAAT}=\frac{{-J}_{NaEAAT}\;z_{Na}\;F\;Vol_{PsECS}}{SA_{PsC}} \end{array}$$

where *J*_*NAEAAT*_ is the Na^+^ flux through the EAAT co-transporter, *z*_*Na*_ is the valency of Na^+^, *F* is the Faradays Constant, Vol_*PsECS*_ is the volume of the perisynaptic ECS, and *SA*_*PsC*_ is the surface area of the PsC.

The associated K^+^ current through the transporter is given by:

$$\begin{array}{l} I_{KEAAT}=\frac{{-I}_{NaEAAT}\;}{3} \end{array}$$

Note that we are not considering glutamate transport to the astrocytic cytosol as we are only interested in Na^+^, K^+^, and Ca^2+^ dynamics. Additionally, our model for EAAT1/2 transport would need further consideration to include the dependency of fluxes on intra and extra cellular ionic concentrations.

#### Sodium Calcium Exchanger (NCX)

The NCX is a reversible antiporter which uses the electrochemical gradient of Na^+^ to exchange 3 Na^+^ ions for 1 Ca^2+^ ion across the membrane. Depending on the membrane potential and transmembrane Na^+^ gradient, the transporter operates either in forward mode (Na^+^ is transported into the cell while Ca^2+^ is extruded) or in the reversed mode (providing for influx of Ca^2+^ and efflux of Na^+^) (Jeffs et al., 2007).

The Na^+^ current component of the transporter is given by Gabbiani and Cox (2010):

$$\begin{array}{l} I_{NaNCX}=({\bar{I}}_{NCX}{\left(\frac{{\left[Na^{+}\right]}_{PsC}}{{\left[Na^{+}\right]}_{PsECS}}\right)}^{3}e\frac{\text{γ}FV}{RT}\\ \;-\left(\frac{{\left[Ca^{2+}\right]}_{PsC}}{{\left[Ca^{2+}\right]}_{PsECS}}\right)e\frac{\left(\gamma -1\right)FV}{RT})\;SA_{PsC} \end{array}$$

where ${\bar{I}}_{NCX}$ is the NCX exchanger conductance and γ is a partition parameter.

The Ca^2+^ current component is given by:

$$\begin{array}{l} I_{CaNCX}=-2\left(\frac{I_{NaNCX}}{3}\right) \end{array}$$

#### Leakage From Perisynaptic ECS to Global ECS

The diffusion of K^+^ and Na^+^ between the PsECS and the GECS is modeled as a simple electrochemical gradient controlled channel in which a zero extracellular potential is assumed and is given by:

$$\begin{array}{l} I_{iECSL}=g_{iECS}E_{iECS}SA_{ECSL} \end{array}$$

where *i* is the ion under consideration, *g*_*iECS*_ is the conductance of the channel, *SA*_*ECSL*_ is the surface area between the PsECS and the GECS, and *Ei*_*ECS*_ is the Nernst like potential of the channel given by:

$$\begin{array}{l} E_{iECS}=\frac{RT}{F}\ln\left(\frac{{\left[i^{+}\right]}_{PsECS}}{{\left[i^{+}\right]}_{GECS}}\right) \end{array}$$

#### Astrocyte Process Ionic Transport Model

Breslin et al. (2018) proposed that ion retention within thin astrocyte processes can give rise to the formation of K^+^ and Na^+^ microdomains at the PsC. This localization of astroglial ionic microdomains arises because in thin processes, surface conduction dominates over volume conduction, and because membrane lipids are negatively charged, deep potential wells form near the dipole heads restricting the flow of cations along the process. Therefore, cations must hop from well to well which restricts ion conduction along the membrane. This hopping effectively semi-isolates the PsC from the astrocytic main body allowing the formation of K^+^ and Na^+^ microdomains at the PsC under different conditions.

Breslin et al. (2018) proposed that the current flow *I*_*iPF*_ (see Figure 2) through the thin process, due to ionic hopping can be represented as:

$$\begin{array}{l} I_{iPF}=K_{i}\frac{{V_{A}-V_{m}-V}_{r}}{l}\\ \exp\left[-\frac{Q_{i}\left(\varphi_{w-}\sqrt{\frac{Q_{i}\left(V_{A}-V_{m}-V_{r}\right)}{l\pi \epsilon }}\right)}{k_{B}T}\right]\;CSA_{P} \end{array}$$

where *K* is a constant which represents mobility and concentration of mobile ions, *V*_*m*_ is the resting membrane potential of the astrocyte, ϕ_*w*_ is the well activation energy or potential barrier to ion flow, *l* is the length of the process, *Q* is the charge on a single ion taken as the charge on an electron, *T* is the absolute temperature, *CSA*_*P*_ is the cross-sectional area of the process, ϵ is the dynamic permittivity and is given by ϵ = ϵ_0_ ϵ_*r*_, where ϵ_0_ is the absolute permittivity, and ϵ_*r*_ is the relative permittivity of the cytoplasm, and k_B_ is the Boltzmann constant.

The concentrations of K^+^, Na^+^, and Ca^2+^ in the astrocyte soma are held constant but will be continuously changing at the PsC thus establishing a dynamic concentration gradient associated with these cations. Consequently, we formulate a Nernst-like reversal potential for Na^+^, K^+^, and Ca^2+^ between the astrocyte soma (AS) and the PsC as:

$$\begin{array}{l} V_{r}=\frac{RT}{F}\ln\left(\frac{{\left[i\right]}_{AS}}{{\left[i\right]}_{PsC}}\right) \end{array}$$

where *i* is the ion under consideration. A more in-depth discussion and assumptions of the full ionic transport mechanism along thin astrocyte processes is given in Breslin et al. (2018).

### Neurone Model

The neuronal model utilized in this work consists of the biophysical Hodgkin and Huxley (HH) type model described in Breslin et al. (2018) with the addition of a voltage-gated Na^+^ channel, K^+^ background channel and an Na^+^ background channel. These background channels reflect implicit influx/efflux pathways, necessary for the system to ensure dynamic equilibrium. All parameter values for the neurone model are described in Table 4. For reasons of simplicity, the internal concentrations of neurone Na^+^ and K^+^ remain constant. Whilst we recognize that the neuron NKA is driven by internal Na^+^ and astrocyte NKA is driven by external K^+^, as we are not considering internal neurone Na^+^ concentration change, we apply the same NKA model for each, altering the maximum pump rates accordingly.

**Table 4:** Neurone parameters.

**Parameter**	**Value**	**Units**	**Description**
P_NKAmaxNeu_	−3.7863 × 10^−8^	mol/m^2^	Maximum NKA-ATPase pump rate
K_NaiNeu_	10 × 10^−3^	M	Na^+^ threshold for NKA-ATPase
K_KENeu_	1.5 × 10^−3^	M	K^+^ threshold for NKA-ATPase
[Na^+^]_Syn_	0.015	M	Na^+^ concentration in the synapse
[K^+^]_Syn_	0.1	M	K^+^ concentration in the synapse
g_KNeu_	360	S/m^2^	Maximum K^+^ channel conductance
g_NaNeu_	1,200	S/m^2^	Maximum Na^+^ channel conductance
g_LNeu_	3	S/m^2^	Maximum leak channel conductance
g_KBNeu_	1.0522	S/m^2^	K^+^ background channel conductance
g_NaBNeu_	2.3217	S/m^2^	Na^+^ background channel conductance
E_KNeu_	−0.12	V	K^+^ channel reversal potential
E_NaNeu_	0.115	V	Na^+^ channel reversal potential
E_LNeu_	0.010613	V	Leak channel reversal potential
C_m_	0.01	F/m^2^	Membrane capacitance

#### Voltage-Gated Neuronal Sodium Channel (Na_*Neu*_)

The HH model simulates current flow of Na^+^ through a voltage-gated channel, therefore the current flow of Na^+^ from the neurone can be modeled as:

$$\begin{array}{l} I_{NaNeu}={-\;g}_{NaNeu}m^{3}\left(V_{Neu}-E_{NaNeu}\right)SA_{Syn} \end{array}$$

where *g*_*NaNeu*_ is the maximum Na^+^ channel conductance, *E*_*NaNeu*_ is the reversal potential of the sodium channel, *V*_*Neu*_ is the membrane voltage of the neurone, and *SA*_*syn*_ is the surface area of the synapse.

#### Neuronal Background Ion Channels (Na_*B*_/K_*B*_)

In this model, there are two individual background ion channels for Na^+^ and K^+^. These channels use the electrochemical gradient between the PsC and ECS, resulting in an influx of Na^+^ and efflux of K^+^ under normal physiological conditions. They were modeled as a simple passive electrochemical gradient dependent channel given by Sterratt et al. (2011):

$$\begin{array}{l} I_{iBNeu}=g_{iBNeu}\left(V_{neu}-E_{i}\right)SA_{Syn} \end{array}$$

where *i* is the ion under consideration, *g*_*iBNeu*_ is the channel conductance. Note: the value of *g*_*iNBeu*_ is chosen in such a way that the total flux of ion *i* = *0* at steady state. *V*_*neu*_ is the neurone membrane voltage, *SA*_*Syn*_ is the surface area of the neuronal synapse, and *E*_*i*_ is the channel Nernst potential and is given by:

$$\begin{array}{l} E_{i}=\frac{RT}{F}\ln\left(\frac{{\left[i^{+}\right]}_{PsECS}}{{\left[i^{+}\right]}_{Syn}}\right) \end{array}$$

Note that the concentrations of K^+^ and Na^+^ within the neuronal synapse are held at baseline.

The complete astrocyte/neurone model was implemented using Matlab 2015b 64-bit (Windows version) by Mathworks. All simulation results presented in the results section of this paper used the forward Euler method of integration with a fixed time step of Δ*t* = 10μs.

## Results

This section reports the outcomes of a series of simulations that demonstrate the formation of a Ca^2+^ microdomain at the PsC. The simulations show that the Ca^2+^ microdomain is a direct result of ion retention along the thin astrocyte process. We have shown in a recent paper (Breslin et al., 2018) that ion retention underpins Na^+^ and K^+^ microdomain formation at the PsC during physiological neuronal excitation. In this work we show that the uptake of Na^+^, via EAAT channels during neuronal stimulus, creates the Na^+^ microdomain in the PsC thereby causing the NCX to reverse with subsequent formation of a Ca^2+^ microdomain in the PsC.

### NCX Reversal Under Physiological Stimulation

To explore the reversal of the astrocyte NCX, a series of simulations were carried out with the presynaptic neurone stimulated using an external current to produce firing rates of 10, 20, and 30 Hz, respectively. The neuronal stimulus has a duration of ~1 min where the first 0.1 min allows the model to reach a steady state condition and the stimulus ceases after 1 min. This long stimulus period allows investigation of what effect sustained neural activity has on the intracellular/extracellular ionic concentrations. In these simulations PsECS Ca^2+^ is held constant at baseline, however K^+^ and Na^+^ are permitted to change via the neurone and astrocyte K^+^ and Na^+^ channels. Each time the neurone spikes, it is assumed that there is 1 mM of glutamate released into the PsC and there are enough EAAT1/2 transports which allow the instantaneous binding of all the extra glutamate. Therefore, the extracellular glutamate concentration always remains at baseline concentration. Furthermore, the maximum flux rate of EAAT1/2 (*J*_0_) is tuned such that 3 mM of Na^+^ is taken up from the PsECs and 1 mM of K^+^ is released into the PsECS by the EAAT1/2 over a period of 30 ms. Moreover, the astrocyte membrane voltage is held constant at ~-80 mV, in line with reported resting membrane potential for astrocytes, which incidentally is close to the reversal potential for the NCX. Therefore, during periods of homeostatic rest there is no net flow of Ca^2+^ or Na^+^ across the membrane associated with the NCX.

**Figure 3:**
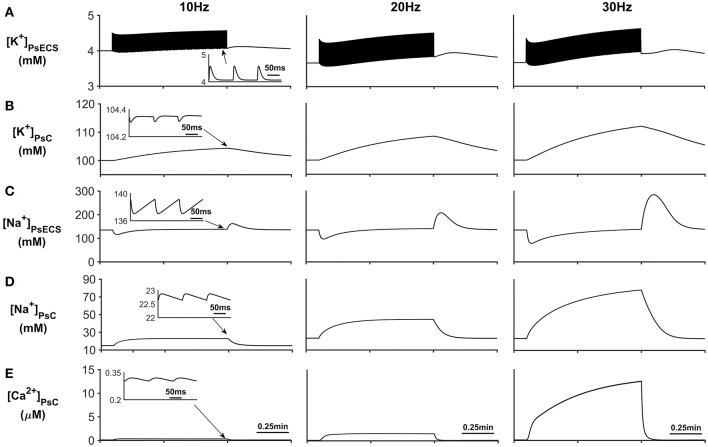
Astrocyte PsC ion concentrations. **(A)** [K^+^]_PsECS_ transient. **(B)** [K^+^]_PsC_ transient. **(C)** [Na^+^]_PsECS_ transient. **(D)** [Na^+^]_PsC_ transient. **(E)** [Ca^2+^]_PsC_ transient. During periods of neuronal activity, increased [K^+^]_PsECS_ is cleared by the astrocyte leading to a microdomain of K^+^ in the PsC. Moreover, due to the influx of Na^+^ predominantly via EAAT channels there is an increase in [Na^+^] PsC, resulting in the reversal of the NCX and Ca^2+^ microdomain formation. The inserts within **(A–E)** show the fast dynamics of the ionic concentration changes within the PsC in response to the neurone activity.

The results presented in Figure 3A show that during periods of neural stimulus, K^+^ ions released by the neurone, build up in the PsECS and are cleared by the astrocyte, which results in a K^+^ microdomain formation at the PsC (Figure 3B). At the start of the neuronal stimulation, there is a transient loss of Na^+^ from the PsECS (Figure 3C), due to neuronal depolarization and astrocytic EAAT transport. Furthermore, the transport of Na^+^ across the astrocyte membrane via Na^+^ background channels and EAAT transporters results in a Na^+^ microdomain formation (Figure 3D).

**Figure 4:**
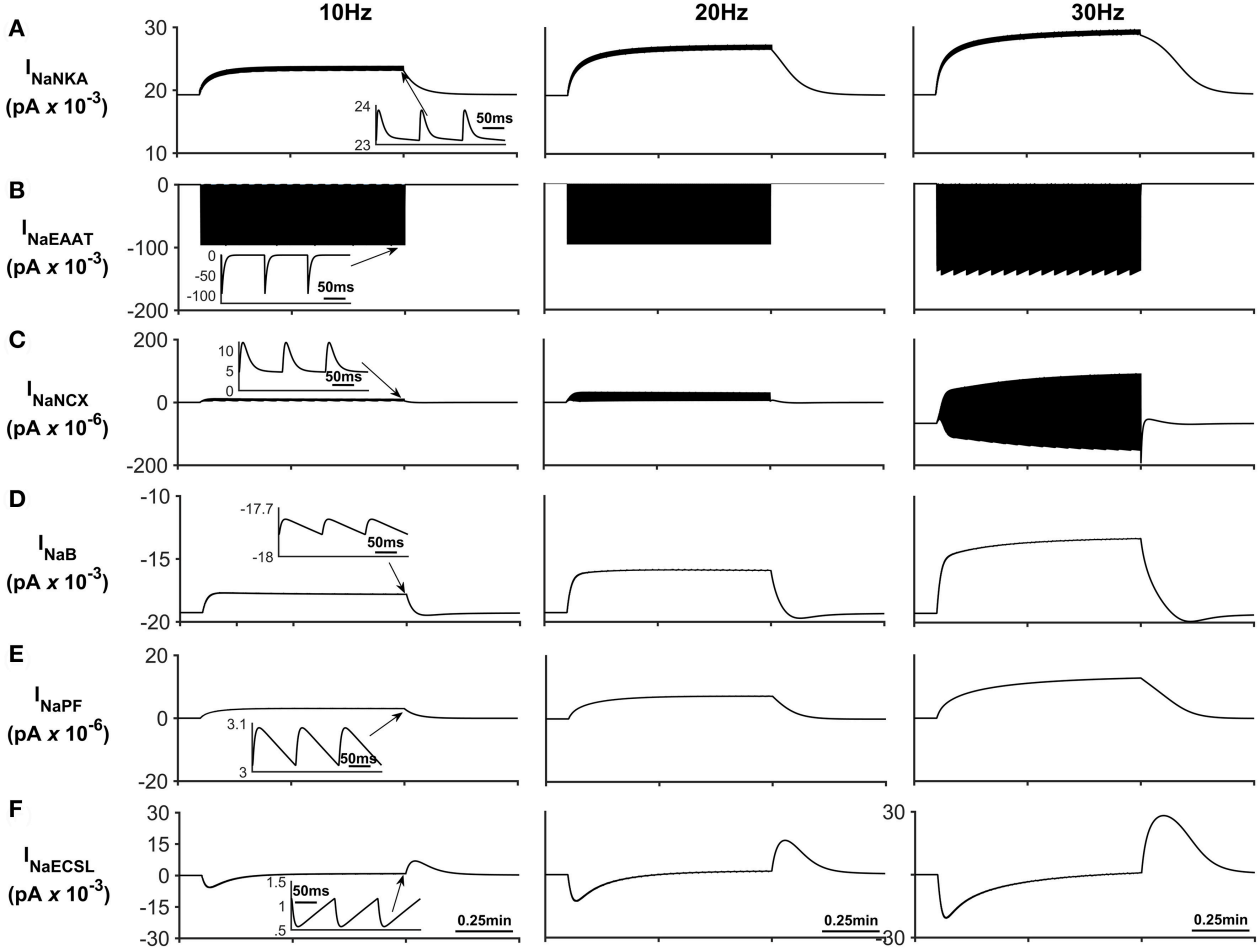
Astrocyte Na^+^ currents. **(A)** Na^+^ NKA current. This current is the main pathway for Na^+^ efflux from the astrocyte is the NKA while the main pathway for sodium efflux is Na^+^ current through the EAAT (see **(B)**) **(C)** Na^+^ NCX current. It can be seen that during periods of neurone stimulation, the NCX reverses which results in the efflux of Na^+^ from the PsC in response to the increased Na^+^ uptake via the EAAT. **(D)** Background Na^+^ current. This represents the uptake of Na^+^ through a lumped model of all other Na^+^ channel. The current can be seen to slow down during periods of PsC Na^+^ increase during neurone stimulus. **(E)** Na^+^ current along the process. The current along the process is governed by the well-hopping mechanism described by Breslin et al. (2018) which severely restricts the flow of Na^+^ from the PsC to the soma. This restriction results in a microdomain of Na^+^ forming in the PsC compartment as the Na^+^ cannot simply diffuse along the process. **(F)** Na^+^ current due to the leakage of Na^+^ from the PsECS to the GECS which is purely diffusive. The inserts within **(A–F)** show the fast dynamics of the currents in response to neurone activity.

In addition to Na^+^ and K^+^ microdomain formation, it is clear from Figure 3E that a local PsC Ca^2+^ microdomain is also formed. This Ca^2+^ microdomain is formed even in the absence of an ER: the ER is widely believed to be essential for astrocyte Ca^2+^ dynamics (Verkhratsky et al., 2012). The microdomain of Ca^2+^ is caused by the reversal of the NCX causing Ca^2+^ influx in exchange for astrocytic Na^+^ efflux. This can be seen in Figure 4C where the only Ca^2+^ influx pathway in our model is via the NCX, the complete reversal of the NCX is due to the sudden changes in PsC Na^+^ during neuronal stimulation onset as the astrocyte membrane voltage is held constant.

Figure 4 presents the Na^+^ currents associated with the astrocyte. The main pathway for Na^+^ efflux from the astrocyte is the NKA (Figure 4A) while the main pathway responsible for Na^+^ uptake is the EAAT (Figure 4B).

The transient loss of Na^+^, observed in the PsECS (Figure 3C), is caused by the large, transient uptake by EAAT1/2 accompanying glutamate removal following neuronal stimulation, and the EAAT remains active for ~30 ms (Figure 4B). This large, transient, EAAT-derived Na^+^ flux, compared to the smaller, slower NKA and NaB activating transport rates, tips the transport uptake/release balance in favor of inward transportation and initiates the formation of a Na^+^ microdomain. As the NKA and NaB uptake increases, due to the increased Na^+^ concentration in the PsC, the influx/efflux, Na^+^ pathways tend toward a state of equilibrium and the microdomain of Na^+^ remains at a stable concentration. It is also noted that as Na^+^ increases in the PsC that the NCX works in reverse mode to remove Na^+^ from the PsC. Figures 4E,F show the Na^+^ currents along the process and between PsECS and GECS, respectively. Since the efflux of Na^+^ via the process is several orders of magnitude smaller than the other Na^+^ currents this is the main driving force for the creation of the Na^+^ microdomain in the PsC. Moreover, as the PsECS Na^+^ concentration changes, the leak current (*I*_*NaECSL*_) between the PsECS and GECS attempts to maintain the Na^+^ levels in the PsECS.

**Figure 5:**
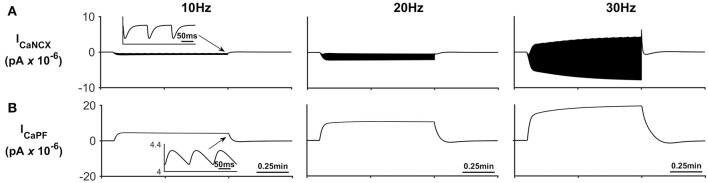
Astrocyte Ca^2+^ currents. **(A)** Astrocyte Ca^2+^ NCX current. During periods of neural stimulation an influx of Na^+^ via the EAAT1/2 cotransporter results in the reversal of the NCX and thus there is an influx of Ca^2+^ into the PsC. **(B)** Ca^2+^ current along the process. The only efflux pathway within the model is along the process. The current along the process is governed by the well-hopping mechanism described by Breslin et al. (2018) which severely restricts the flow of Ca^2+^ from the PsC to the soma. This restriction results in a microdomain of Ca^2+^ forming in the PsC compartment. The inserts within **(A,B)** show the fast dynamics of the currents in response to neurone activity.

Figure 5 describes the astrocytic Ca^2+^ currents; again, it can be seen that during neural stimulation, the influx of Na^+^ cause the NCX to work in reverse mode and therefore there is an influx of Ca^2+^ (Figure 5A). The only efflux pathway for Ca^2+^ considered within the model is via the thin astrocyte process, which is also governed by the well-hopping mechanism described in Breslin et al. (2018) (Figure 5B), Therefore the efflux pathway is much less dominant than the influx pathway which results in a microdomain of Ca^2+^ forming, as seen in Figure 3E.

**Figure 6:**
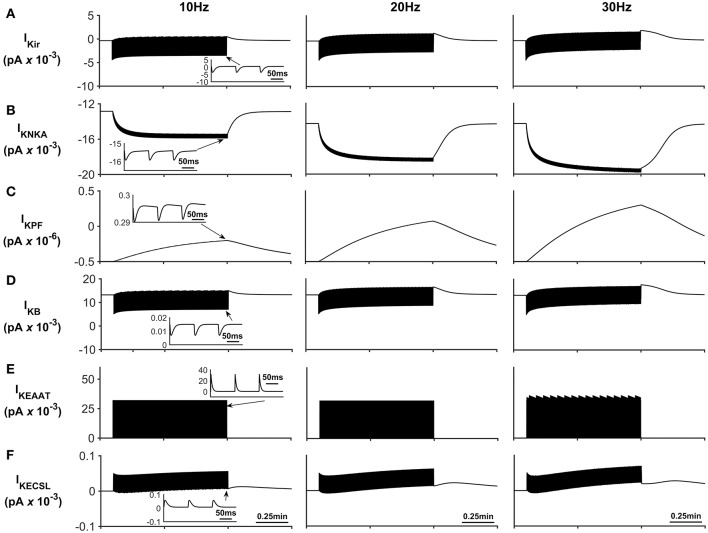
Astrocyte K^+^ currents. **(A)** K^+^ Kir Current. During periods of neurone stimulation the Kir channel will initially uptake K^+^ before releasing it again at a much slower rate (see insert **(A)**). **(B)** K^+^ NKA current. The rate of NKA increases with neurone stimulation due to the increase of PsECS K^+^. This current is mainly responsible for the uptake K^+^ in the PsC. **(C)** K^+^ current along the process. Since the current along the process is governed by the well hopping mechanism described by Breslin et al. (2018) it severely restricts the flow of K^+^ from the PsC to the soma. This restriction results in a microdomain of K^+^ forming in the PsC compartment as the K^+^ cannot simply diffuse along the process. Furthermore, K^+^ is transported across the membrane via background and EAAT K^+^ currents (**(D,E)**, respectively) much more slowly as these currents are mainly driven by transient K^+^ fluctuations in the PsECS due to the neurone stimulation; while these currents have a higher peak magnitude, they are much more “spike like in nature” than NKA currents (See inserts **(B,D,E)**). **(F)** K^+^ current due to the leakage of K^+^ from the PsECS to the GECS which is purely diffusive.

The K^+^ currents behave in a similar manner as previously described in Breslin et al. (2018). NKA and Kir represent the dominant fluxes of K^+^ across the astrocytic membrane; NKA is purely responsible for K^+^ influx whereas Kir is in a constant state of transition between forward (K^+^ influx) and reverse (K^+^ efflux) mode during neuronal stimulus (see Figure 6). When neural stimulus ceases, the voltage-dependent reversal of Kir, along with efflux of K^+^ via the K^+^ background channel, brings the PsC levels of K^+^ back to the initial resting state.

## Discussion

Intracellular ionic signaling represents the substrate for glial excitability (Verkhratsky and Nedergaard, 2018). These intracellular signaling events are mediated through spatially and temporally organized fluctuations in the concentration of major ions; there is firm evidence for physiologically relevant Ca^2+^ and Na^+^ signaling (Rose and Verkhratsky, 2016; Verkhratsky and Nedergaard, 2018; Verkhratsky et al., 2019), Cl^−^ signaling (Wilson and Mongin, 2018; Verkhratsky et al., 2019), and K^+^ signaling (Rimmele and Chatton, 2014; Olsen et al., 2015). The formation of localized concentration microdomains is critical for ionic signaling spatial fidelity; while the mechanisms underlying formation of these microdomains remain under debate, a new hypothesis has already been proposed (Breslin et al., 2018). In the CNS, most excitatory synapses are tightly enwrapped by perisynaptic astroglial processes forming the synaptic cradle (Reichenbach et al., 2010; Verkhratsky and Nedergaard, 2014). This structure provides homeostatic control of the synaptic cleft and therefore requires the ability to generate relevant signals in response to neuronal activity. The perisynaptic astroglial compartment is devoid of the ER (Reichenbach et al., 2010), hence excluding the metabotropic pathway for generation of local Ca^2+^ microdomains. Astrocytes express glutamatergic ionotropic receptors, although the receptor-mediated current density is rather low (with whole cell currents rarely exceeding 10–100 pA), thus limiting the ionic influx (Verkhratsky and Burnstock, 2014; Rusakov, 2015). Astroglial perisynaptic membranes also express high densities of glutamate transporters (EAAT1/2), which couple glutamate transport with substantial Na^+^ influx (Kirischuk et al., 2007; Langer and Rose, 2009). These transporters are co-localized with NCX (Minelli et al., 2007), which couple Na^+^ and Ca^2+^ fluxes in opposite directions. Here we applied the reduced model of PsC to test the hypothesis that glutamate transporters and NCX working together are sufficient to create local Ca^2+^ microdomains in astroglial perisynaptic cradles.

Our model demonstrates that stimulation of astrocytes with glutamate, mimicking neuronal activity, generates substantial Na^+^ influx, which forms local microdomains due to the previously suggested mechanism of ion retention, where cation retention in wells dominates over conventional electrochemical diffusion (Breslin et al., 2018; Wade et al., 2018). Moreover, the generation of Ca^2+^ microdomains has previously been reported, albeit not at the fine process level, but the underlying ionic fluxes (and channels/transporters contributing to) have not been examined (Rusakov, 2015). This computational modeling study has identified the molecular targets and their relative contributions to the formation of a Ca^2+^ microdomain in the absence of an ER region. Specifically, our model predicts that the generation of a Na^+^ microdomain switches the NCX into reverse mode, which is sufficient to produce relevant focal Ca^2+^ signals; while cross-disciplinary research to test this hypothesis is beyond the scope of this paper, we envisage that this work will instigate such a project.

In essence only the EAAT, NKA, and NCX are necessary and sufficient for the development of a Ca^2+^ microdomain: note that to avoid rapidly increasing Ca^2+^ concentrations within the cradle, with increasing neuronal frequency, our model would require a Ca^2+^ efflux pathway, and a likely candidate is the PMCA pump. These mechanisms, which do not depend on intracellular sources for Ca^2+^, directly link neuronal activity and glutamate release to the formation of Na^+^ and Ca^2+^ microdomains in the perisynaptic astroglial processes, instrumental for generation of astroglial homeostatic response, which is critical for maintenance of synaptic transmission.

## Author Contributions

JW, LM, AV, MD, SH, HV, BF, and AB contributed to the conception and design of the study. JW and KB developed the software for simulations. JW and BF created all graphics and data visualizations. JW, KB, LM, AV, MD, and AB wrote the first draft of the manuscript. JW, LM, AV, MD, SH, HV, BF, AB, JH, KW-L, and KB contributed to manuscript revision, read and approved the submitted version.

### Conflict of Interest Statement

The authors declare that the research was conducted in the absence of any commercial or financial relationships that could be construed as a potential conflict of interest.
